# Prognostic significance of the dynamic changes of systemic inflammatory response in metastatic renal cell carcinoma

**DOI:** 10.1590/S1677-5538.IBJU.2017.0500

**Published:** 2019

**Authors:** Beihe Wang, Weijie Gu, Fangning Wan, Guohai Shi, Dingwei Ye

**Affiliations:** 1 Department of Urology, Fudan University Shanghai Cancer Center, Shanghai, China;; 2 Department of Oncology, Shanghai Medical College, Fudan University, Shanghai, China

**Keywords:** Carcinoma, Renal Cell, Molecular Targeted Therapy, Prognosis

## Abstract

**Purpose:**

To elucidate the prognostic value of systemic inflammatory response in patients with metastatic renal cell carcinoma (mRCC) who are treated with sunitinib, we evaluated the prognostic role of C-reactive protein (CRP) kinetics. This study also compared prognostic models containing CRP kinetics and neutrophil-to-lymphocyte ratio (NLR) kinetics.

**Materials and Methods:**

A consecutive cohort of 94 patients with mRCC who were treated with sunitinib was retrospectively included from Fudan University Shanghai Cancer Center. According to dynamic changes in CRP and the NLR, patients were divided into three groups for analysis of CRP and NLR kinetics. The associations between survival and potential prognostic factors were assessed. The incremental value of prognostication was evaluated.

**Results:**

A significant difference (P<0.001) in overall survival (OS) was observed among the three groups of CRP kinetics. The median OS of the non-elevated group was nearly 1.3-fold longer than that of the normalized group (33.0 vs. 26.3 months), and two times longer than that of the non-normalized group (33.0 vs. 14.0 months). Multivariate analysis showed that CRP and NLR kinetics were independent prognostic indicators. The model containing CRP kinetics had a better predictive accuracy than that with NLR kinetics, which was supported by the C-index (0.731 vs. 0.684) and the likelihood ratio χ^2^ test (79.9% vs. 44.9%).

**Conclusion:**

Our study suggests that dynamic changes in CRP can better predict survival in patients with mRCC who are treated with sunitinib. Routine assessment of CRP before and after targeted therapy would help identify patients at risk of a poor outcome.

## INTRODUCTION

Renal cell carcinoma (RCC), which accounts for 2-3% of adult malignancies (1), is the third most common urogenital malignancy in China. Despite progress in diagnosis of RCC, especially using abdominal imaging, 20-30% of all patients are initially diagnosed with metastatic RCC. Additionally, among the 70-80% of patients with disease confined to the kidney, approximately 20% of them experience systemic relapse and develop metastatic RCC (mRCC) after curative nephrectomy (2). The outcome of these patients has greatly improved because of a dramatic shift in the management of patients with mRCC from cytokines to molecular-targeted therapy in the last decade (3-5). However, the major cost and toxicity accompanied by targeted therapy have resulted in the need for new prognostic indicators to better stratify patients and select therapies (6).

Inflammation is a hallmark of cancer, which is often accompanied by inflammatory cell infiltration and activated stroma (7). Therefore, several prognostic biomarkers based on circulating blood cells have been developed to predict patient's outcome in various tumors. Among these biomarkers, growing evidence has shown that C-reactive protein (CPR) and the neutrophil-to-lymphocyte ratio (NLR) are associated with a poor prognosis in RCC (8, 9). While baseline CRP levels have been shown to be associated with prognosis of patients with mRCC, CRP kinetics are also of prognostic value (10). Saito et al. showed that CRP kinetics could better predict overall survival (OS) by improving the predictive accuracy by 4% compared with baseline CRP levels alone in patients with mRCC who were treated with multimodal therapy (11). However, most of this evidence was found in the era of cytokine therapy. With the emerging of targeted therapy, the influence of NLR kinetics was evaluated by Templeton et al. in patients with mRCC (12), but they didn't compare it with the other inflammation marker. Therefore, the importance of CRP as a prognostic indicator and comparison between these models should be re-evaluated in the era of targeted therapy.

Based on these considerations, we previously studied patients with mRCC who were treated by sunitinib (13). Our previous study provided useful insight into the long-term safety and efficacy of sunitinib for treating these patients. The present study aimed to evaluate the prognostic role that CRP kinetics play in patients with mRCC who are treated with sunitinib. We also aimed to compare models containing CRP kinetics and NLR kinetics.

## MATERIALS AND METHODS

### Patients

A consecutive cohort of 94 histologically confirmed RCC patients with clinically proven metastasis who were treated with sunitinib were retrospectively studied between February 2008 and July 2014. A trained study nurse collected data on the patient's clinical characteristics, laboratory data, treatment, and follow-up information. The institutional review board of Fudan University Shanghai Cancer Center approved the study protocol and the study was carried out according to the approved guidelines. Each patient was well informed about the details of this study and informed consent was obtained.

The eligibility criteria were as follows: (1) age of 18 years or older; (2) clinically proven metastatic clear cell renal carcinoma; and (3) sunitinib was used as either as first-line or second-line therapy. Other inclusion criteria included a complete blood routine test, measurement of serum CRP levels at pre-treatment and during the treatment, Eastern Cooperative Oncology Group performance status of less than or equal to 2, normal renal, hepatic, and bone marrow function, and absent or stable central nervous system metastasis. Of the 94 patients, nine were excluded for the absence of serum CRP levels during the treatment.

Responses and progression were assessed by a professional radiologist according to the Response Evaluation Criteria in Solid Tumors (14). OS was defined as the time from initiation of treatment to the date of death or last contact. Progression-free survival (PFS) was defined as the time from initiation of treatment to the date of progression. A routine measurement of serum CRP levels was performed before initiating treatment, and at the end of the 1st, 2nd, and 3rd months after starting treatment. CRP levels were also measured at some time points when the patient's condition changed during the first 3 months.

### Statistical analysis

Categorical data are presented as frequencies and percentages, and continuous data as means and interquartile range.

For analysis of CPR kinetics, the patients were divided into three groups according to baseline CRP levels and changes in CRP levels as previously reported as follows (15): (1) patients whose baseline CRP levels were <5.3mg/L (non-elevated group); (2) patients whose baseline CRP levels were ≥5.3mg/L, but normalized at least one time during the first 3 months (normalized group); and (3) patients whose CRP levels never decreased to a normal level (non-normalized group). The threshold of CRP was determined by receiver operating characteristics with the highest sensitivity, which was set at 5.3mg/L.

Calculation of NLR kinetics was based on the baseline NLR and changes in the NLR by 12 weeks (±2 weeks), calculated as % change ([NLR at week 12/baseline NLR]-1X100). These patients were subsequently divided into three groups as follows: (1) increased group (>50% increase); (2) stable group (<50% decrease to <50% increase); and (3) decreased group (>50% decrease).

The Memorial Sloan-Kettering Cancer Center (MSKCC) model includes Karnofsky performance status <80%, serum lactate dehydrogenase levels >1.5 times the upper limit of normal, an interval from diagnosis to treatment of <1 year, corrected serum calcium levels greater than the upper limit of normal, and serum hemoglobin levels less than the lower limit of normal (16). This model assigns patients into favorable (no risk factor), intermediate (one or two risk factors), and poor (more than two risk factors) according to the number of risk factors predicting poor outcomes.

Distributions of OS and PFS were estimated using the Kaplan-Meier method in different groups of CRP and NLR kinetics.

Associations between endpoints and potential prognostic factors were assessed by using the log-rank test in univariate analysis. The Cox proportional hazards model was subsequently used in multivariate analysis to assess the independent effect of the variables. Hazard ratios (HRs) and 95% confidential intervals (CIs) of covariates were calculated. The predictive accuracy of the two Cox models was evaluated by Harrell's concordance index (C-index). The likelihood ratio χ^2^ test was used to assess whether CRP kinetics or NLR kinetics added predictive value to the baseline models.

R software was applied for all statistical analyses. The level of statistical significance was set at P<0.05 and all P values were two-sided.

## RESULTS

### Patient's characteristics

The demographic characteristics of the patients and biochemical factors are shown in Table-1. Of the 85 patients available for analysis, 58 (62.2%) were men and 27 (37.8%) were women, with a median age of 58 years (interquartile range, 48-63 years). Twenty-nine (34.1%) patients scored 0 points in the MSKCC, 49 (57.6%) scored 1 point, and seven (8.2%) scored 2 points. The most common organ of metastasis was the lung (n=78), followed by bone (n=24) and liver (n=17). Median baseline and nadir CRP levels were 6mg/L (interquartile range, 1.5-8.7mg/L) and 4.5mg/L (interquartile range, 2.0-6.2mg/L), respectively. Thirty-four (40%) patients whose baseline CRP levels were <5.3mg/L were assigned to the non-elevated group. In 26 (30.6%) of the 51 patients with elevated baseline CRP levels, nadir CRP levels normalized to <5.3mg/L, and they were assigned to the normalized group. The non-normalized group consisted of 25 (29.4%) patients whose baseline CRP levels were ≥5.3mg/L and nadir CRP levels never normalized. With regard to NLR kinetics, the majority of patients were assigned to the stable group (n=48, 56.5%), 20 (23.5%) were assigned to the increased group, and 17 (20%) were assigned to the decreased group.

**Table 1:** Patient's Characteristics.

Variables		N (%)
**Age**		
	Median(IQR)	58 (48-63)
**Gender**		
	Male	58 (68.2)
	Female	27 (31.8)
**Treatment regimen**		
	First-line	9 (10.6)
	Second-line	76 (89.4)
**Surgery**		
	Nephrectomy	73 (85.9)
**Pathology**		
	Clear cell carcinoma	78 (91.7)
	Clear cell carcinoma with sarcomatoid differentiation	3 (3.5)
	Papillary carcinoma	2 (2.4)
	Unclassified*	2 (2.4)
**Baseline CRP**		
	Median (IQR)	6.0 (1.5-8.7)
**Nadir CRP**		
	Median (IQR)	4.5 (2.0-6.2)
**CRP kinetics**		
	Non-normalized group	25 (29.4)
	Normalized group	26 (30.6)
	Non-elevated group	34 (40.0)
**Baseline NLR**		
	Median (IQR)	3.0 (2.2-4.0)
**NLR kinetics**		
	Increased	20 (23.5)
	Stable	48 (56.5)
	Decreased	17 (20)
**Hemoglobin**		
	Median (IQR)	128.0 (113.5-144.0)
**Albumin**		
	Median (IQR)	40.6 (37.8-43.8)
**Corrected calcium**		
	Median (IQR)	8.4 (8.2-8.8)
**LDH**		
	Median (IQR)	155.0 (129.5-190.5)
**Karnofsky performance status**		
	≥80%	62 (72.9)
	<80%	23 (27.1)
**Time from diagnosis to sunitinib treatment**		
	<12 months	55 (64.7)
	≥12 months	30 (35.3)
**MSKCC**		
	Favourable	29 (34.1)
	Intermediate	49 (57.6)
	Poor	7 (8.2)
**Number of metastatic sites**		
	1	58 (68.2)
	≥2	27 (31.8)
**Metastatic sites**		
	Lung	59
	Liver	5
	Bone	23
	Brain	1
	Lymph node	14
	Abdomen	16
	Skin	4
**Overall survival, months**		
	Median (IQR)	21.9 (12.3-47.9)
**Progression free survival, months**		
Median (IQR)		10.8 (5.5-33.2)

**IQR** = interquartile range; **CRP** = C-reactive protein; **NLR** = neutrophil-to-lymphocyte ratio; **LDH** = lactate dehydrogenase; **MSKCC** = Memorial Sloan-Kettering Cancer Center.

* Diagnosed with clear cell carcinoma after consultation of pathology.

### Survival analysis

The median follow-up time was 28.6 months (95% CI: 18.2-36.2). Median OS and PFS of the patients were 21.9 (95% CI: 12.3-47.9) and 10.8 months (95% CI: 5.5-33.2).

**Figure 1:**
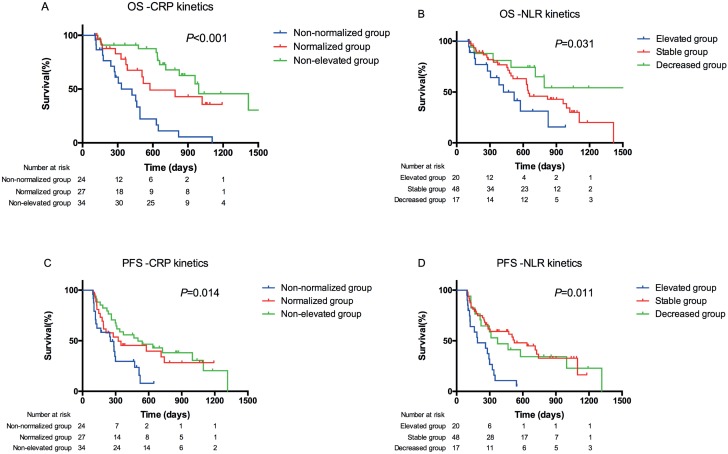
Overall survival (OS) and progression-free survival (PFS) analysis of patients with metastatic renal cell carcinoma based on CRP and NLR kinetics. Kaplan-Meier analysis of OS for CRP kinetics (A) and NLR kinetics (B), and PFS for CRP kinetics (C) and NLR kinetics (D).

Kaplan-Meier curves of different groups for CRP and NLR kinetics for OS and PFS were created (Figures 1A-D). These curves showed a significant difference in overall OS between the three groups of CRP kinetics (P<0.001, Figure-1A) and NLR kinetics (P=0.031, Figure-1B). The median OS of the non-elevated group was nearly 1.3-fold longer than that of the normalized group (33.0 vs. 26.3 months), and two times longer than that of the non-normalized group (33.0 vs. 14.0 months).

In univariate analysis, CRP and NLR kinetics were associated with prognosis (Table-2). For CRP kinetics, the normalized and non-normalized groups were associated with a poorer prognosis compared with the non-elevated group. The MSKCC score and the number of metastatic organs (P=0.001 for OS; P=0.001 for PFS) were significantly associated with OS and PFS. Baseline CRP levels (≥5.3 vs. <5.3mg/L, P=0.002) achieved significance only when analyzed with OS.

**Table 2:** Univariate Analysis.

Variables		OS			PFS		
		HR	95% CI	P	HR	95% CI	P
**Gender**							
	Male	Ref.			Ref.		
	Female	0.799	0.409-1.559	0.510	0.785	0.44-1.399	0.411
	Age	1.010	0.982-1.038	0.489	0.991	0.969-1.013	0.427
**Baseline CRP**							
	<5.3	Ref.			Ref.		
	≥5.3	2.845	1.472-5.500	0.002	1.575	0.926-2.681	0.094
	Nadir CRP	1.027	0.984-1.071	0.227	1.071	0.981-1.053	0.363
**CRP kinetics**							
	Non-normalized group	Ref.			Ref.		
	Normalized group	0.291	0.136-0.622	0.001	0.433	0.220-0.851	0.015
	Non-elevated group	0.196	0.095-0.405	<0.001	0.393	0.209-0.738	0.004
	Baseline NLR	0.894	0.754-1.06	0.197	0.933	0.810-1.075	0.337
**NLR kinetics**							
	Increased group	Ref.			Ref.		
	Stable group	0.557	0.271-1.141	0.110	0.329	0.178-0.610	<0.001
	Decreased group	0.265	0.095-0.743	0.012	0.369	0.173-0.789	0.010
	Hemoglobin	0.985	0.969-1.002	0.085	0.992	0.977-1.006	0.258
	Albumin	0.953	0.897-1.012	0.116	0.947	0.899-0.998	0.043
	Corrected calcium	1.000	1.000-1.000	0.986	1.000	1.000-1.000	0.929
	LDH	0.999	0.995-1.003	0.503	1.000	0.999-1.002	0.768
K**arnofsky performance status**							
	≥80%	Ref.			Ref.		
	<80%	2.505	1.296-4.839	0.006	2.341	1.321-4.148	0.004
**Time from diagnosis to sunitinib treatment**							
	<12 months	Ref.			Ref.		
	≥12 months	2.436	1.321-4.492	0.004	1.435	0.835-2.467	0.192
**MSKCC**							
	0	Ref.			Ref.		
	1	2.207	1.093-4.456	0.027	1.328	0.736-2.397	0.346
	2	4.596	1.598-13.222	0.005	3.903	1.609-9.466	0.003
**Number of metastatic organs**							
	1	Ref.			Ref.		
	≥2	2.836	1.541-5.220	0.001	2.463	1.433-4.234	0.001

**CRP** = C-reactive protein; **NLR** = neutrophil-to-lymphocyte ratio; **LDH** = lactate dehydrogenase; **MSKCC** = Memorial Sloan-Kettering Cancer Center

**Figure 2:**
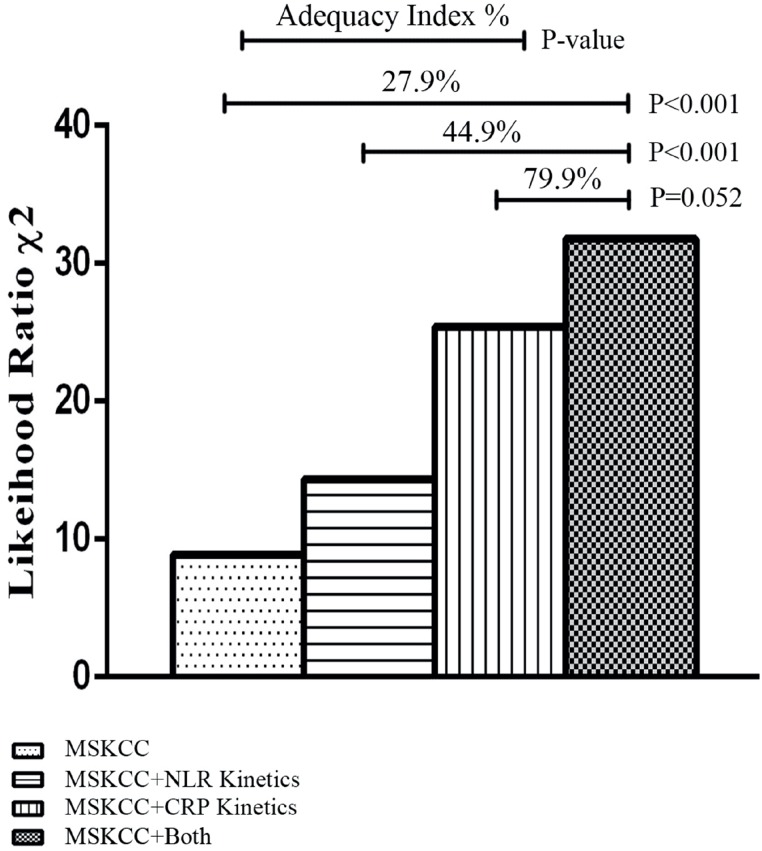
The likelihood ratio χ^2^ test showing the adequacy index for different prognostic models. The columns, from left to right, show the adequacy indices for MSKCC, MSKCC+CRP kinetics, MSKCC+NLR kinetics, and MSKCC+CRP kinetics+CRP kinetics.

In multivariate analysis, a full Cox proportional hazards model was built considering CRP and NLR kinetics separately (Table-3). The CRP model included age, sex, MSKCC, number of metastatic organs and CRP kinetics, while the NLR model replaced CRP kinetics with NLR kinetics. Age and sex were excluded from the final models for insignificant P values. The reduced models showed that CRP and NLR kinetics were independent prognostic factors, as was the number of metastatic organs and MSKCC. The predictive accuracy of the two models was evaluated by the C-index, which was 0.731 (95% CI: 0.542-0.919) for the CRP model and 0.684 (95% CI: 0.502-0.867) for the NLR model. The likelihood ratio χ^2^ test showed that the adequacy index was improved by 79.9% in the CRP model, and this was 35.0% higher than that in the NLR model (Figure-2).

**Table 3:** Multivariate Analysis for Overall Survival.

Variables	Category	Full model	Reduced model		Full model			Reduced model	
		P	HR	95% CI	P	P	HR	95% CI	P
Age	continuously coded	0.749	—	—	—	0.821	—	—	—
Gender	Female vs. male	0.713	—	—	—	0.378	—	—	—
Number of metastatic organs	1 vs. ≥2	0.034	1.999	1.042-3.834	0.037	0.003	2.546	1.377-4.775	0.003
MSKCC	0	Ref.	Ref.			Ref.	Ref.		
	1	0.198	1.648	0.811-3.349	0.167	0.124	1.740	0.848-3.572	0.131
	2	0.012	3.886	1.330-11.349	0.013	0.017	4.258	1.411-12.852	0.010
CRP kinetics	Non-normalized group	Ref.	Ref.						
	Normalized group	0.015	0.374	0.167-0.837	0.017				
	Non-elevated group	0.001	0.271	0.128-0.574	0.001				
NLR kinetics	Increased					Ref.	Ref.		
	Stable					0.038	0.476	0.225-1.006	0.052
	Decreased					0.023	0.316	0.110-0.903	0.032
C-index(95%CI)			0.731 (0.542-0.919)				0.684 (0.502-0.867)		

**MSKCC** = Memorial Sloan-Kettering Cancer Center; **CRP** = C-reactive protein; **NLR** = neutrophil-to-lymphocyte ratio; **C-index** = Harrell's concordance index

## DISCUSSION

In the present study, we performed complete analysis of dynamic changes in inflammatory biomarkers, which were shown to be associated with OS in patients with metastatic RCC who were treated with sunitinib. Additionally, this study validated the prognostic model proposed by Motzer et al. (16), as well as the prognostic model containing NLR proposed by Templeton et al. Previous studies on the effect of inflammatory biomarkers can be grouped into two types. One type focuses on pretreatment baseline levels and the other focuses on dynamic changes. The effects of baseline CRP levels and the NLR as prognostic factors have been well determined in this era of targeted therapy. The dynamic change in these inflammatory markers is still yielding for further studies. Teishima et al. first reported the prognostic value of change in CRP for mRCC patients treated with targeted agents (17, 18). However, they failed to examine the predictive ability of the model containing CRP and compare it with other inflammatory markers. The present study further validated the prognostic value of CRP kinetics and is the first to compare prognostic models containing the kinetics of two major inflammatory biomarkers. We showed for the first time that CRP kinetics could better stratify patients with mRCC under targeted therapy compared with NLR kinetics.

The exact mechanism of the inflammatory response to cancer is still unknown. However, there is a large amount of evidence showing that the inflammatory process involves underlying mechanisms that relate to progression of cancer. Cancer-associated inflammation may actually share a similar pathway and some aspects of their contribution to tumor development, including disease progression, malignant conversion, invasion, and metastasis (19). To date, it seems to be associated with a combination of relative neutrophilia and lymphopenia and production of major inflammatory cytokines, such as interleukin-6 (IL-6) (20, 21). Neutrophilia has been observed in RCC (22), and vascular endothelial growth factor secreted by neutrophils plays an important part in angiogenesis (23). Together with IL-6, which is a representative molecule in inflammatory responses during progression of RCC (24), these growth factors modulate the tumor environment to improve its growth and dissemination (25). Moreover, lymphopenia with a decrease in CD4+T-cells, which are often found in patients with cancer, will compromise the antitumor response mediated by lymphocytes (26, 27). Furthermore, CRP levels can be markedly elevated in the early phase under stimulation of pro-inflammatory cytokines, such as IL-6 (28). Taken together, these findings suggest that CRP and the NLR can be used for prognostication of RCC.

To investigate the applicability of CRP and the NLR in the daily clinical setting, we identified the optimal cut-off as a dichotomous variable. A cut-off of 5.3mg/L, classifying patients with a baseline CRP, conferred the highest prognostic value. Using this cut-off, we found a strong association of CRP kinetics with survival in patients who were treated with targeted therapy. This finding was previously reported in patients who were treated with immunotherapy (15). We also showed that NLR kinetics were strongly associated with survival outcomes, which is similar to previous reports (12). However, the value of the NLR count failed to be a significant predictor of survival. Moreover, whether the value of the NLR count is determined by relative lymphopenia or an increase in myeloid cells is unclear.

In addition to established first- and second-line molecular-targeted therapies, immunotherapeutic agents, such as checkpoint inhibitors, have demonstrated feasibility in many solid tumors, including RCC (29, 30). The checkpoint blockade blocks the interaction of checkpoint receptors on immune cells and inhibitory ligands on tumor cell to induce immune responses at different levels, including the upregulation of proinflammatory cytokine expression (30-32). However, the association between inflammatory markers and prognosis of mRCC patients treated with checkpoint inhibitors is still unclear, awaiting future work in this area.

We acknowledge a number of limitations in our study. As an unplanned analysis, these results need to be validated in prospective studies. Moreover, inflammatory markers including CRP and NLR could be influenced by some confounding factors that we currently lack information on, such as allergy, infection, metabolic syndrome and the use of non-steroidal anti-inflammatory drugs, within this subset. Notably, the cut-offs of the NLR and CRP were selected based on their prognostic value in our dataset. The optimal cut-off of inflammatory biomarkers should be further investigated in large samples and needs to be validated in prospective studies.

## CONCLUSIONS

In conclusion, our study supports the hypothesis that inflammation plays a role in the prognosis of renal cancer. Baseline CRP levels and CRP kinetics have emerged as important biomarkers in mRCC because of their association with OS. Our findings suggest that routine assessment of CRP levels before and after targeted therapy would help to better identify patients at risk of poor outcomes.

## ABBREVIATIONS

RCC: = renal cell carcinoma
mRCC: = metastatic RCC
CPR: = C-reactive protein
NLR: = neutrophil-to-lymphocyte ratio
OS: = overall survival
PFS: = progression-free survival
MSKCC: = Memorial Sloan-Kettering Cancer Center
HRs: = Hazard ratios
CIs: = confidential intervals
C-index: = Harrell's concordance index
